# Assessing mesophotic coral ecosystems inside and outside a Caribbean marine protected area

**DOI:** 10.1098/rsos.180835

**Published:** 2018-10-31

**Authors:** Erika Gress, Maria J. Arroyo-Gerez, Georgina Wright, Dominic A. Andradi-Brown

**Affiliations:** 1 Conservation Leadership Programme, David Attenborough Building, Pembroke Street, Cambridge CB2 3QZ, UK; 2 Nekton Foundation, Begbroke Science Park, Begbroke Hill, Woodstock Road, Begbroke, Oxfordshire OX5 1PF, UK; 3 Operation Wallacea, Wallace House, Old Bolingbroke, Spilsby, Lincolnshire PE23 4EX, United Kingdom; 4 Ocean Conservation, World Wildlife Fund - US, 1250 24th St NW, Washington, DC 20037, USA; 5 Department of Zoology, University of Oxford, South Parks Road, Oxford OX1 3PS, UK

**Keywords:** mesophotic coral ecosystems, Cozumel, Mexico, marine protected areas, deep reef conservation, deep reef refugia

## Abstract

Widespread shallow coral reef loss has led to calls for more holistic approaches to coral reef management, requiring inclusion of ecosystems interacting with shallow coral reefs in management plans. Yet, almost all current reef management is biased towards shallow reefs, and overlooks that coral reefs extend beyond shallow waters to mesophotic coral ecosystems (MCEs; 30–150 m). We present the first detailed quantitative characterization of MCEs off Cozumel, Mexico, on the northern Mesoamerican Reef in the Mexican Caribbean, and provide insights into their general state. We documented MCE biodiversity, and assessed whether MCEs adjacent to a major town and port, where coastal development has caused shallow reef damage, have similar benthic and fish communities to MCEs within a National Park. Our results show that overall MCE communities are similar regardless of protection, though some taxa-specific differences exist in benthic communities between sites within the MPA and areas outside. Regardless of protection and location, and in contrast to shallow reefs, all observed Cozumel MCEs were continuous reefs with the main structural habitat complexity provided by calcareous macroalgae, sponges, gorgonians and black corals. Hard corals were present on MCEs, although at low abundance. We found that 42.5% of fish species recorded on Cozumel could be found on both shallow reefs and MCEs, including 39.6% of commercially valuable fish species. These results suggest that MCEs could play an important role in supporting fish populations. However, regardless of protection and depth, we found few large-body fishes (greater than 500 mm), which were nearly absent at all studied sites. Cozumel MCEs contain diverse benthic and fish assemblages, including commercially valuable fisheries species and ecosystem engineers, such as black corals. Because of their inherent biodiversity and identified threats, MCEs should be incorporated into shallow-reef-focused Cozumel National Park management plan.

## 1. Introduction

Coral reef ecosystems border nearly a sixth of global coastlines [1], have high biodiversity [2] and play a crucial food security role for millions of people [3]. Economic value of the ecosystem services provided by coral reefs has been estimated to be higher than any other ecosystem in our planet, about 350 000 Intl. dollar per year per hectare of coral reefs [4]. However, reef ecosystems face widespread threats, both from local scale impacts (e.g. overfishing and pollution) and from large scale ones (e.g. coral bleaching and ocean acidification) [1,3,5]. In the face of such threats, many recent conservation efforts have focused on maintaining reef resilience [6] combining the ability of reefs to both resist stressors and recover from damage following impact [7].

Mesophotic coral ecosystems (MCEs, reefs approx. 30–150 m) have received considerable attention because of the postulate that they could serve as deep refuges and support degraded shallow reefs [8,9]. Studies show that upper-MCEs (30–60 m) can share species with shallow reefs [8–15], though lower-MCEs (60–150 m) often comprise deeper-water specialist species [10–12,16,17]. Hard corals (Scleractinian) and fish connectivity between MCEs and adjacent shallow reefs appears to be species specific [18,19]. In addition, recent studies show that MCEs are also impacted by anthropogenic factors [17,20], jeopardizing our opportunities to understand their diversity, biological traits and ecosystem services. The role of MCEs in supporting overall reef resilience needs to be better understood.

MCEs remain under-studied and poorly integrated into reef management plans compared to shallow reefs, because of technical, logistical and financial challenges associated with accessing them. Several examples of MCEs being integrated into broader reef management exist. In Eilat (Gulf of Aqaba, Red Sea) following MCE documentation, an existing marine park boundary was moved to 500 m further offshore, and to 50 m depth to incorporate MCEs into the protected area [9,21]. Other MCE areas, such as the *Oculina* reefs off the Florida coast, have received fisheries protection through establishment of a new marine protected area (MPA) after surveys indicated the damage caused by trawling in the area [20,22,23]. In Colombia, a national park has been set up to protect MCE and deep-sea (greater than 200 m) habitats [24]. Even with limited MCE data, it is possible to integrate MCEs into MPAs. For example, MCEs became incorporated within the Great Barrier Reef management plan by ensuring representation of different geological seabed features when conducting park zonation [25]. These approaches fit with recently advocated holistic reef management, considering the ecosystems interacting with shallow coral reefs in management plans [9].

This study provides the first detailed quantitative benthic and fish community characterization of MCEs off Cozumel, Mexico. Cozumel is an island located 17 km off the east coast of the Yucatan peninsula, at the northern extent of the Mesoamerican Reef [26]. There, extensive fringing coral reef ecosystems off the west coast of Cozumel are well recognized for their biological and socioeconomic importance [27–31]. Cozumel's reefs are heavily visited by recreational SCUBA divers, and reef-related tourism constitutes a major component of the island and the region's economy. In 2015, the port of Cozumel received 3.8 million passengers that arrived on 1240 vessels [32]. Cozumel has also supported a renowned black coral jewellery industry since the early 1960s. Extensive black coral populations were reported on shallow reefs (less than 30 m) and MCEs from 30 to 80 m, but overexploitation happened prior to the first ecological surveys and harvest regulation implementation [27,28,33,34], leaving few shallow and upper-MCE colonies remaining [30,35]. This highlights the need for studies at all reef depths, to increase our understanding of current reef state, and to ensure adequate protection.

**Figure 1.**
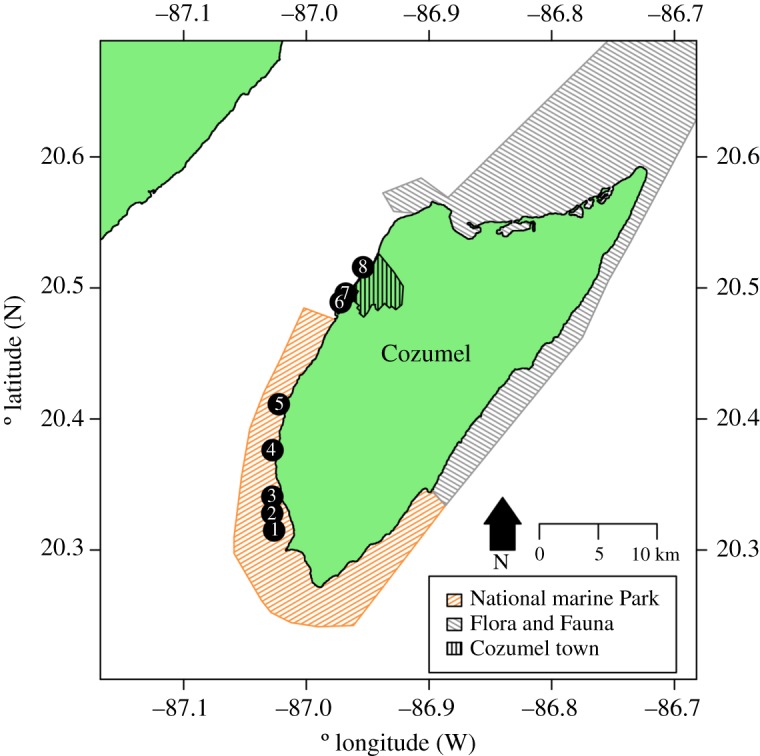
Location of survey sites relative to Cozumel and the National Park and Flora and Fauna Protected Areas on Cozumel. Both shallow reefs and MCEs were surveyed at each site. Sites and their approximate distances from the main development in parenthesis were 1, Colombia (24.9 km); 2, Herradura (23.3 km); 3, Palancar Jardines (22.6 km); 4, Santa Rosa (18.2 km); 5, Punta Tunich (14.2 km); 6, Villa Blanca (4.1 km); 7, Transito Transbordador (3.3 km) and 8, Purgatorio (0.6 km). All distances from the main development were measured from the passenger ferry terminal in the centre of town following the edge of the reef crest in Google Earth. GPS locations for sites are given in electronic supplementary material, S1.

The reefs of Cozumel are under two protection regimes: Parque Nacional Arrecifes de Cozumel (Cozumel National Park) in the southwest, and the Flora and Fauna Protected Area in the north and east coasts (figure 1). Cozumel National Park was decreed in 1996, is approximately 120 km^2^ in area, and is zoned for multiple use types [36]. The management plan states that this MPA extends to the 100 m depth isobath [36], although it contains no acknowledgement of occurrence or management strategies for MCEs. The MPA is zoned, with each zone having different regulations, but most allow SCUBA diving, scientific research and other tourism activities (including sport fishing). Hook and line fishing is allowed in some non-intensive use zones (e.g. where few tourism activities occur) [36]. Cozumel shallow reefs within the National Park (henceforth MPA or protected area) are considered in ‘very good’ condition, because they have regionally high scleractinian corals coverage (20–40%) [37]. The Flora and Fauna Protected Area was designated in 2012 and is also zoned. Fisheries activities are allowed in the Flora and Fauna Protected Area, although it has a core zone of 4.7 km^2^ that is fully no-take [38]. The majority of Cozumel reefs are contained within one of these two protection schemes, with the only area of reef without any protected status adjacent to the main town (figure 1). Here, the development of cruise and ferry vessel terminals and tourism infrastructure adjacent to the reef is known to have caused widespread shallow reef degradation [29,39]—including declines in hard coral cover from 44% to 4% over the period 1995–2005 [39]—though the impact on MCEs is unknown.

In this study, we investigate how key benthic groups and fish communities, on both shallow reefs and MCEs, differ between the established MPA and heavily impacted sites adjacent to the main town and cruise port [29,39]. We specifically document shallow reef and MCE ecological communities and assess whether MCEs—by virtue of their depth—naturally provide protection from coastal anthropogenic disturbance. If this were the case, we would expect unprotected MCE communities adjacent to the main town to be ecologically similar to protected MCEs, despite the disturbance causing differences in shallow reef condition. We would also expect ecological community differences, between shallow reefs and adjacent MCEs, to vary based on whether the shallow reefs are highly impacted or not. We provide the first detailed quantitative characterization of MCEs off western Cozumel, Mexico. These data could serve as a baseline for future studies, and also provide insight into current shallow reef and MCE state.

## 2. Methods

### 2.1. Reef surveys

Surveys were conducted at eight sites on the west side of Cozumel between 15 and 30 August 2016. Five sites were within the Cozumel National Park (MPA), and three were adjacent to Cozumel town and port with no protection (non-MPA). The MPA sites were (1) Colombia, (2) Herradura, (3) Palancar Jardines, (4) Santa Rosa and (5) Punta Tunich, while the non-MPA sites were (6) Villa Blanca, (7) Transito Transbordador and (8) Purgatorio (figure 1; electronic supplementary material, S1). At each site, we surveyed both the shallow reef (15 m) and MCEs (55 m), completing four transects at each depth, giving a total of eight transects per site, and 64 transects in total in this study. Transects were 30 m in length and each separated by a 10 m interval. Each transect was surveyed for both benthic and fish communities, and all surveys were conducted using open-circuit SCUBA equipment between the hours of 7.00 and 11.00.

Benthic surveys were conducted along the same transect lines following the fish surveys (see below), using a GoPro Hero 4 Black camera. Transect lines were marked at 2.5 m intervals, and a planar photo quadrat was taken at the start and then at every 2.5 m intervals along the transect, including the end, giving 13 quadrats per transect. When taking quadrats, the camera was held perpendicular to the reef at approximately 0.4 m above the benthos.

Fish surveys were conducted using a diver-operated stereo-video system (stereo-DOV), consisting of two cameras separated by 0.8 m and with approximately 3° convergence angle filming forward along the reef (see [40] for system overview). The stereo-DOV system records two synchronized images of reef fish, allowing accurate measurements of fish length. The stereo-DOV used two GoPro Hero 4 Black cameras and a spool system with biodegradable line for measuring out each transect. GoPro cameras are considered appropriate for reef fish length measurements, and perform similarly to other camera systems [41]. At the beginning of the dive, the stereo-DOV operator started the cameras recording and synchronized them using a torch which was turned on and off repeatedly by the dive buddy. The cameras were then pointed downwards while the buddy attached the end of the biodegradable line to the reef. The stereo-DOV operator swam with the cameras down, reeling out the line, until the first marker was reached after 10 m of line. At this point, the cameras were pointed forward along the reef to record the transect. After reaching the marker indicating a further 30 m of line had been unreeled, the cameras were pointed back down for 10 m before starting the next transect. This was repeated over 4 transects, with all transect start and endpoints, and transect intervals pre-marked on the biodegradable line.

### 2.2. Video/image analysis

Benthic photos were analysed using Coral Point Count with Excel extensions (CPCe) [42] to determine the percentage cover of different benthic categories. Ten random points were placed on each quadrat image in CPCe, and the substrate category at each point was identified. The total number of points of each substrate category per transect was then used to calculate benthic percentage coverage for each transect. Categories were: black coral (Antipatharia), hard coral (Scleractinia), calcareous macroalgae, fleshy macroalgae, turf algae, crustose coralline algae, sponge, gorgonian, hydrozoan (specifying *Millepora*), cyanobateria and non-living substrate.

The stereo-DOV footage was analysed using EventMeasure (v. 4.42, SeaGIS, Melbourne, Australia). Transects were synchronized, and all fish 2.5 m either side of the camera (5 m transect width by 5 m height; constrained using EventMeasure) were identified to species, or the lowest taxonomic level possible and measured from snout to the tip of caudal peduncle. From the length and species identification, the biomass was estimated based on length–weight ratios from Fishbase [43], based on the equation: *W* = *aL^b^* where *W* is the weight, *L* is the length and *a* and *b* are given parameters for a specific species.

### 2.3. Data analysis

To evaluate differences in percentage coverage of key benthic groups between MPA and non-MPA sites, a Mann–Whitney *U* test was used on mean percentage cover of each benthic group at each depth. To test for broader differences in benthic community assemblage based on depth, protection (inside or outside the MPA), and interactions between these factors, permutational multivariate analysis of variance (PERMANOVA) was used on Bray–Curtis dissimilarities of percentage cover of all benthic categories [44]. Bray–Curtis is appropriate to be used for percentage cover in PERMANOVA when there are zeros recorded for some benthic categories [44]. As our sites are situated along the west coast of Cozumel, to control for any potential natural variation along this coastline, we fitted latitude as a fixed effect in our PERMANOVA. To further explore differences in benthic community structure based on protection at different depths, redundancy analyses were conducted using the function ‘rda’ in vegan [45]. This redundancy analysis was based on removing non-living substrate and recalculating the percentage community composition based on the proportion of all living components of the community. Non-living benthic cover was removed to standardize for differences in reef type between depths. Two separate redundancy analyses were run, using Euclidian distances for the shallow reef and MCE data separately, using the formula ‘Benthic Percentage Composition ∼ Protection + Site’. Protection was a categorical variable, representing whether a site was within or outside the MPA.

Differences in overall fish species richness, total fish biomass and commercially valuable fish biomass were identified using ANOVA fitting depth and protection as factors. Commercially valuable fish species were identified based on Fishbase [43] fisheries price category classification of medium, high or very high. Model residual plots and Q-Q plots were inspected to ensure that the data met the assumptions of ANOVA (normality of residuals, homogeneity of variance). Models were simplified to remove non-significant factors or interactions based on minimizing the Akaike information criterion (AIC). To identify patterns in fish community, we used three multivariate statistical techniques based on the Bray–Curtis dissimilarities calculated from the fourth root transformed species biomass. Firstly, to visualize differences in the fish communities, we used non-metric multidimensional scaling (NMDS) constrained to two axes. Secondly, to statistically test for the effects of protection and depth, we used PERMANOVA including latitude and site as factors. Thirdly, to identify fish species that may be driving observed patterns in community structure, we conducted a principal coordinate analysis (PCOA). We extracted the first two PCOA axes and tested the biomass of each fish species for correlations with either axis. Any fish species with a Pearson's correlation coefficient |*r*^2^| > 0.4 was identified. The biomass of these species was then tested using a permutational-ANOVA for significant differences based on protection, depth or the interaction between protection and depth. We followed Langlois *et al*. [46] to use kernel density estimates to compare length distributions between fish surveyed within and outside the MPA. Bandwidths were selected using the Sheather–Jones selection procedure [47] within the ‘dpik’ function in the ‘KernSmooth’ package [48]. Differences in the length distributions were then tested using the permutational ‘sm.density.compare’ function in the R package ‘sm’ [49]. This function randomly allocates recorded fish lengths between MPA and non-MPA sites, and then calculates how different our observed data were from this randomization across the length distribution.

All permutational-ANOVAs and PERMANOVAs were fitted using the ‘adonis’ function in vegan [45] and run for 99 999 permutations. The ‘adonis’ function uses Type 1 (sequential) sums of squares, which means that each term is sequentially fitted after taking account of the previously fitted terms. This approach allowed us to test for effects of protection and depth after controlling for latitude. All analyses were conducted in R [50]. All raw data are contained in electronic supplementary material, S2–S6, and R code for analysis in electronic supplementary material, S7.

## 3. Results

### 3.1. Benthic communities

**Figure 2.**
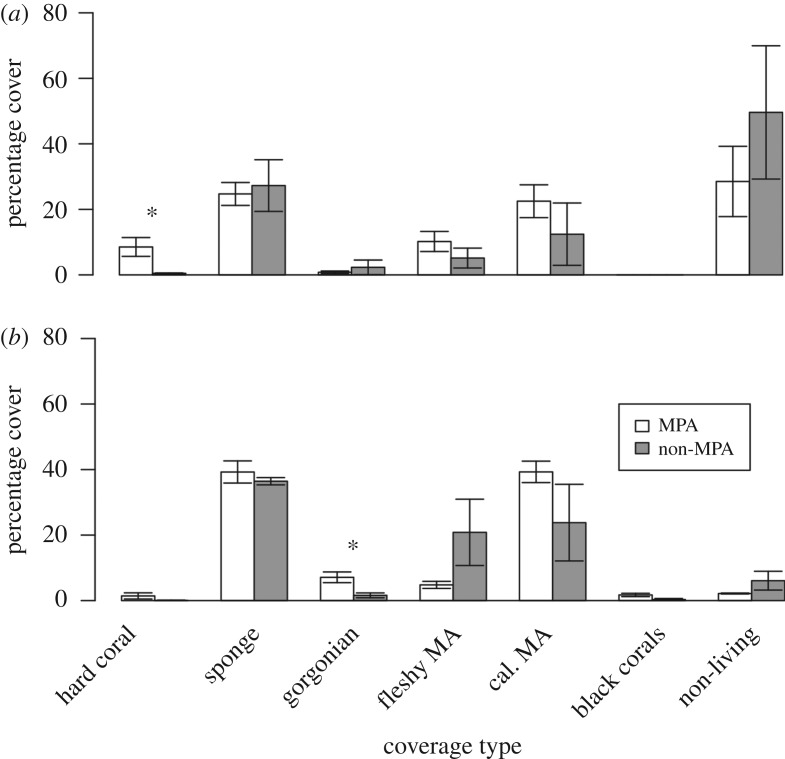
Percentage cover of broad benthic groups on (*a*) shallow reefs at 15 m and (*b*) MCEs at 55 m around Cozumel. Error bars represent one standard error. Significantly different coverage (*p* < 0.05) between protected and unprotected areas was tested using a Mann–Whitney *U* test and indicated with a ‘*’.

We identified differences in benthic communities based on depth, but also a significant interaction between protection and depth indicating that the difference between shallow reefs and MCEs is affected by whether sites are inside or outside the MPA (table 1). Benthic communities also varied between sites and with latitude (table 1). We found higher hard coral cover on shallow reefs inside the MPA (8.5 ± 2.9% cover; mean ± s.e.) than outside (0.5 ± 0.1%), and higher gorgonian coverage on MCEs inside the MPA (7.1 ± 1.6%) than outside (1.6 ± 0.7%) (figure 2). No other significant differences were detected between percentage cover of major groups such as sponges, macroalgae (MA) and non-living substrate between areas of the same depth based on protection (figure 2). There were major differences in benthic cover between shallow reefs and MCEs (table 1 and figure 2), with all surveyed Cozumel MCEs existing as continuous reef systems dominated by sponges and calcareous macroalgae (mostly *Halimeda*), with black corals present and very little of the benthos covered by non-living substrates (figure 2*b*). By contrast, the shallow reefs of Cozumel were characterized by areas of reef separated by patches of sand resulting in higher non-living benthic cover (figure 2*a*). A full list of hard coral and black coral species identified at each depth is contained in electronic supplementary material, S8.

**Table 1.** Benthic PERMANOVA testing for differences in benthic community structure between different protection types, depths and sites and the interactions between them. Model was of the form: benthic matrix ∼ latitude + protection × site × depth.

source	d.f.	mean square	pseudo-F	*p-*value
latitude	1	0.7	12.4	<0.001
protection	1	0.1	0.9	0.467
site	1	0.3	6.1	<0.001
depth	6	0.5	9.8	<0.001
protection : depth	1	0.4	8.0	<0.001
site : depth	5	0.2	3.7	<0.001
residuals	48	0.1		
total	63

**Figure 3.**
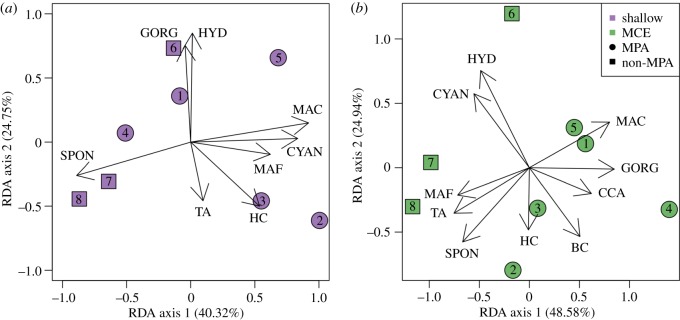
Redundancy analysis of the benthic coverage data standardized to remove non-living benthic cover for (*a*) shallow reefs at 15 m, and (*b*) MCEs at 55 m. Variation explained by each axis is indicated in parenthesis on the axis' label. The length and direction of the arrows corresponds to increasing cover of benthic categories at sites located in that region of the plot. Benthic categories were: BC, black coral; CCA, crustose coralline algae; CYAN, cyanobacteria; GORG, gorgonian; HC, hard coral; HYD, hydrozoan; MAC, calcareous macroalgae; MAF, fleshy macroalgae; SPON, sponge and TA, turf algae.

In the shallows, we found that two of our three sites outside the MPA were correlated with higher sponge cover, while the other site outside the MPA had higher gorgonian and hydroid cover (figure 3*a*). The highest shallow reef hard coral cover was associated with two of the MPA sites, Palancar Jardines and Herradura, at 15.7 ± 6.9% and 14.4 ± 2.3% cover, respectively. In addition to being associated with higher hard coral cover in the shallows, both Palancar Jardines and Herradura were associated with higher hard coral cover on MCEs (figure 3*b*), with Herradura having the highest hard coral coverage we observed on Cozumel MCEs at 5.1 ± 2.0%. The three sites outside the MPA had the lowest hard coral cover at 0.6 ± 0.6% (Purgatorio), 0.2 ± 0.2% (Transito Transbordador) and 0.6 ± 0.4% (Villa Blanca). On MCEs, sites inside the MPA were associated with higher gorgonian, black coral and crustose coralline algae cover (figure 3*b*). Black corals were recorded at all five MCEs within the MPA, but only at Purgatorio MCE outside the MPA. However, overall recorded black coral coverage was low, with 3.0 ± 1.2% at Palancar Jardines and 2.9 ± 2.9% at Santa Rosa, the two sites with the greatest coverage.

### 3.2. Fish communities

**Figure 4.**
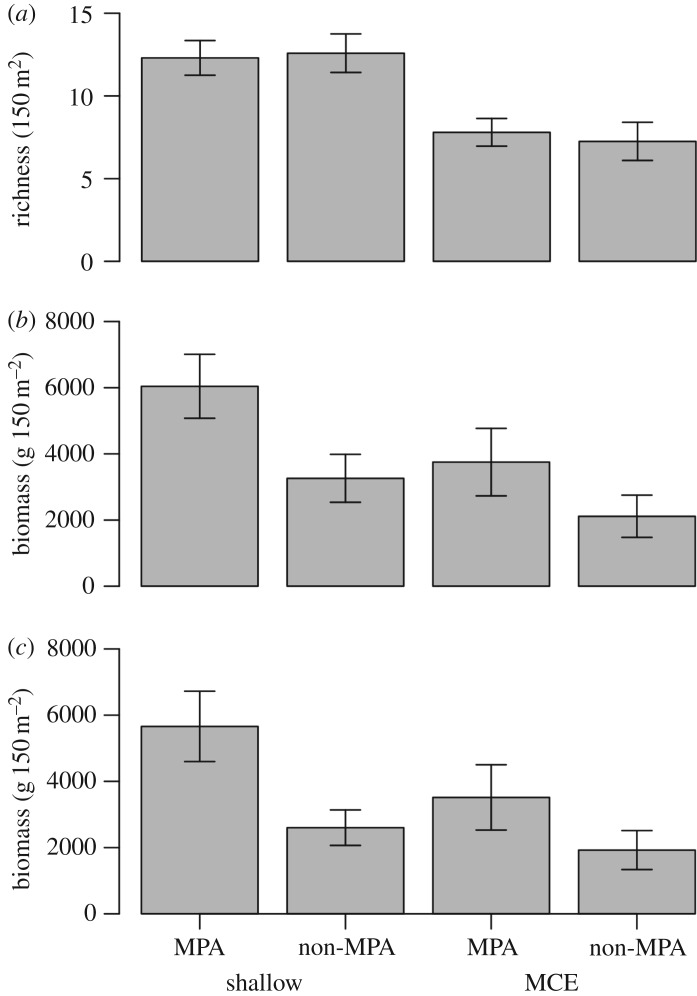
Comparisons of reef fish for shallow reefs (15 m) and MCEs (55 m) for (*a*) species richness, (*b*) all fish biomass and (*c*) commercially important fish biomass. Error bars indicate one standard error. (*a*) Significant effects of depth were found on overall fish species richness, but no effect of depth was found. (*b,c*) Significant effects of protection were detected, but no effect of depth was found.

No difference in fish species richness was identified between shallow reefs located inside and outside the protected area or between MCEs located inside and outside the protected area (figure 4*a*). However, fish species richness was higher on shallow reefs than MCEs (*F*_1,13_ = 22.8, *p* < 0.001), with a mean shallow reef fish species richness of 12.4 ± 0.7 species per 150 m^2^ in contrast to 7.6 ± 0.6 mean species richness per 150 m^2^ on MCEs. Overall, we recorded 80 fish species on Cozumel reefs in this study, with 39 species (48.8%) only recorded on shallow reefs, seven species (8.9%) only recorded on MCEs and 34 species (42.5%) recorded on both shallow reefs and MCEs. The full list of which species were recorded at one or both depths is available in electronic supplementary material, S9. From the 80 fish species recorded, 53 fish species were considered of ‘medium’, ‘high’ or ‘very high’ commercial value in Fishbase. Of these commercially valuable fish, 26 species were found on shallow reefs only (49.1%), six species (11.3%) were found on MCEs only and 21 species (39.6%) were at both depths. Therefore, from the depth-restricted species we recorded, 66.7% of shallow-only fish species and 85.7% of the MCE-only fish species were commercially valuable.

We detected higher fish biomass regardless of depth associated with sites within the MPA for overall fish biomass (*F*_1,13_ = 5.1, *p* = 0.04; figure 4*b*) and commercially important fish biomass (*F*_1,13_ = 5.5, *p* = 0.04; figure 4*c*). We found no significant interaction between depth and protection (so removed this interaction from the model during simplification) or effect of depth (shallow versus MCE) on overall fish biomass (*F*_1,13_ = 3.9, *p* = 0.07; figure 4*b*) or commercially valuable fish biomass (*F*_1,13_ = 2.8, *p* = 0.12; figure 4*c*). However, during model simplification for both overall fish biomass and commercially valuable fish biomass, we found that removing depth from the model resulted in a higher model AIC value than retaining it, suggesting that differences with depth may affect reef fish biomass.

**Figure 5.**
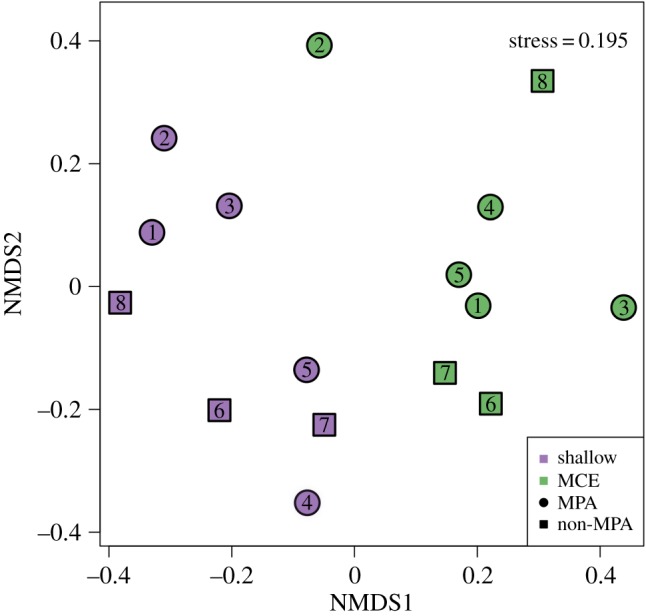
NMDS plot of fish community composition. Species-level fish biomass data was fourth root transformed before Bray–Curtis dissimilarities were calculated.

When considering the fish community structure, and after controlling for the significant effect of latitude, we found effects of depth, and an interaction between protection and depth (table 2). This implies that differences in the fish community between the MPA and non-MPA areas were affected by depth. We visualized the fish community using NMDS (figure 5), which suggested that depth was a more influential factor of reef fish community structure than protection. To identify which fish species might be driving these patterns, we conducted a PCOA and calculated the Pearson's correlation coefficient (*r*^2^) between each individual fish species biomass and the first two PCOA axes. In total, we found 30 fish species that correlated |*r*^2^| > 0.4 with the PCOA axes, of which eight were commercially valuable (table 3). Of the 30 species, eight had significant depth ∶ protection interactions for biomass; all, with the exception of *Xanthichthys ringens*, were small bodied non-commercially valuable species. These depth : protection interactions were driven by three broad patterns (table 3). Firstly, *Stegastes adustus* and *Stegastes diencaeus* both had higher biomass on shallow reefs inside the MPA than on shallow reefs outside and were absent/near-absent from MCEs. Secondly, *Xanthichthys ringens, Chaetodon striatus, Thalassoma bifasciatum* and *Abudefduf saxatilis* had higher biomass on unprotected shallow reefs and were also rare or absent from MCEs. Thirdly, *Chromis cyanea* and *Halichoeres garnoti* ratio of biomass between MPA and non-MPA sites varied based on depth. Additionally, the biomass of six other fish species was affected by depth (table 3). *Prognathodes aculeatus* and *Chromis insolata* were absent/near-absent from shallow reefs, but present on MCEs, while *Acanthurus bahianus*, *Acanthurus coeruleus, Scarus iseri* and *Sparisoma viride* were present on both shallow reefs and MCEs, but with greater biomass in the shallows. Therefore, the biomass of 14 of the 30 fish species correlating with the PCOA axes was affected by solely depth or depth ∶ protection interactions (table 3).

**Table 2.** Fish PERMANOVA testing for differences in fish community structure between different protection types, depths and sites and the interactions between them. Model was of the form: fish matrix ∼ latitude + protection × site × depth.

source	d.f.	mean square	pseudo-F	*p*-value
latitude	1	0.9	4.6	<0.001
protection	1	0.2	0.8	0.658
site	6	0.6	3.3	<0.001
depth	1	1.9	9.9	<0.001
protection : depth	1	0.5	2.7	0.002
site : depth	5	0.3	1.7	0.001
residuals	48	0.2		
total	63

**Table 3.** Biomass of fish species correlating |*r*^2^| > 0.4 with the PCOA from inside versus outside the MPA, and shallow reefs versus MCEs. Depth and protection effects were tested using a permutational-ANOVA including latitude and site, with significant effects (*p* < 0.05) highlighted in italics. Model was of the form: species matrix ∼ latitude + protection × site × depth.

family/sub-family/species	commercially valuable	shallow reefs				MCEs				depth effect		protection effect		depth : protection interaction	
		MPA		non-MPA		MPA		non-MPA							
		mean biomass (g 150 m^−2^)	s.e.	mean biomass (g 150 m^−2^)	s.e.	mean biomass (g 150 m^−2^)	s.e.	mean biomass (g 150 m^−2^)	s.e.	pseudo-F	*p-*value	pseudo-F	*p*-value	pseudo-F	*p*-value
**Acanthuridae**															
*Acanthurus bahianus*	X	39.7	26.0	102.3	102.3	0.0	0.0	6.9	6.9	9.4	*<0.001*	0.1	0.822	0.7	0.464
*Acanthurus coeruleus*	X	526.1	169.6	839.9	431.5	107.5	30.0	67.9	34.4	20.5	*<0.001*	1.3	0.261	1.8	0.196
**Balistidae**															
*Xanthichthys ringens*	X	23.3	23.3	114.1	26.0	76.8	45.1	46.0	9.4	0.0	0.955	2.7	0.108	5.2	*0.025*
**Chaetodontidae**															
*Chaetodon capistratus*		15.1	5.1	71.5	46.5	18.3	9.2	21.1	11.5	4.0	0.047	2.8	0.095	2.7	0.109
*Chaetodon striatus*		0.0	0.0	15.7	7.9	0.2	0.2	0.0	0.0	2.4	0.127	1.1	0.312	3.0	*0.044*
*Prognathodes aculeatus*		0.0	0.0	0.0	0.0	1.1	0.8	1.6	1.3	5.0	*0.024*	1.7	0.206	0.4	0.537
**Haemulidae**															
*Haemulon carbonarium*	X	22.3	15.0	51.4	51.4	0.0	0.0	0.0	0.0	1.7	0.223	0.0	0.957	0.6	0.533
*Haemulon macrostomum*	X	0.0	0.0	0.0	0.0	0.0	0.0	21.0	21.0	1.0	0.875	0.0	1.000	1.6	0.125
*Haemulon melanurum*	X	309.0	309.0	271.1	165.4	0.0	0.0	0.0	0.0	0.4	0.524	2.4	0.106	1.4	0.295
*Haemulon plumierii*	X	13.9	13.9	7.0	7.0	0.0	0.0	27.3	27.3	0.0	0.949	0.0	0.964	1.6	0.305
*Haemulon sciurus*	X	42.5	26.0	32.5	23.5	0.0	0.0	0.0	0.0	1.4	0.234	0.6	0.483	0.0	0.913
**Labridae**															
*Halichoeres garnoti*		17.5	7.3	95.7	55.8	1.4	0.6	4.9	3.9	32.6	*<0.001*	1.9	0.171	20.7	*<0.001*
*Halichoeres maculipinna*		0.2	0.2	0.5	0.5	0.0	0.0	2.2	2.2	0.4	0.848	0.0	0.923	1.2	0.344
*Halichoeres pictus*		0.5	0.4	0.6	0.6	0.0	0.0	0.0	0.0	3.2	0.073	0.9	0.377	0.0	0.917
*Thalassoma bifasciatum*		12.7	2.9	34.9	14.5	1.0	0.9	0.8	0.8	20.2	*<0.001*	0.1	0.801	5.2	*0.024*
**Lutjanidae**															
*Lutjanus analis*	X	62.4	62.4	61.6	61.6	0.0	0.0	0.0	0.0	0.2	0.855	1.0	0.340	0.6	0.522
*Ocyurus chrysurus*	X	210.6	117.1	0.0	0.0	64.3	64.3	0.0	0.0	0.9	0.372	1.1	0.309	0.8	0.415
**Pomacanthidae**															
*Holacanthus tricolor*	X	0.0	0.0	33.7	23.6	5.3	5.3	19.8	10.0	0.1	0.821	1.2	0.284	1.0	0.355
*Pomacanthus arcuatus*	X	84.0	84.0	0.0	0.0	375.0	229.8	1067.3	539.1	2.6	0.110	0.3	0.626	1.8	0.192
**Pomacentridae**															
*Abudefduf saxatilis*		44.1	38.4	107.2	107.2	0.0	0.0	0.0	0.0	37.4	*<0.001*	0.3	0.576	5.4	*0.019*
*Chromis cyanea*		123.7	37.4	7.6	6.2	31.0	10.9	15.2	13.0	6.5	*0.012*	0.3	0.628	7.4	*0.007*
*Chromis insolata*		0.4	0.4	0.0	0.0	73.1	24.1	64.3	22.5	18.3	*<0.001*	0.1	0.768	0.0	0.892
*Stegastes adustus*		15.7	10.9	0.0	0.0	1.2	1.2	4.0	4.0	5.8	*0.012*	5.4	*0.021*	9.9	*<0.001*
*Stegastes diencaeus*		14.9	7.9	2.4	2.4	0.0	0.0	0.0	0.0	20.0	*<0.001*	0.5	0.474	9.6	*0.002*
*Stegastes planifrons*		1.4	0.9	0.6	0.6	0.0	0.0	0.0	0.0	0.4	0.695	0.5	0.513	0.0	0.956
**Scaridae**															
*Scarus iseri*	X	108.1	52.7	61.0	31.4	52.4	24.1	14.7	14.7	4.2	*0.046*	0.1	0.815	0.2	0.689
*Sparisoma aurofrenatum*	X	124.6	42.0	288.4	231.0	106.7	46.1	133.3	81.1	4.1	0.044	1.1	0.313	0.7	0.424
*Sparisoma viride*	X	389.6	254.1	234.9	135.4	0.0	0.0	23.0	23.0	6.5	*0.003*	1.9	0.175	0.8	0.431
**Serranidae**															
*Epinephelus adscensionis*	X	4.6	4.6	12.3	12.3	0.0	0.0	0.0	0.0	2.2	0.146	0.3	0.625	0.3	0.630
**Tetraodontidae**															
*Canthigaster rostrata*		3.1	1.3	11.0	2.4	79.4	42.8	8.2	4.2	2.6	0.068	0.0	0.981	2.3	0.141

**Figure 6.**
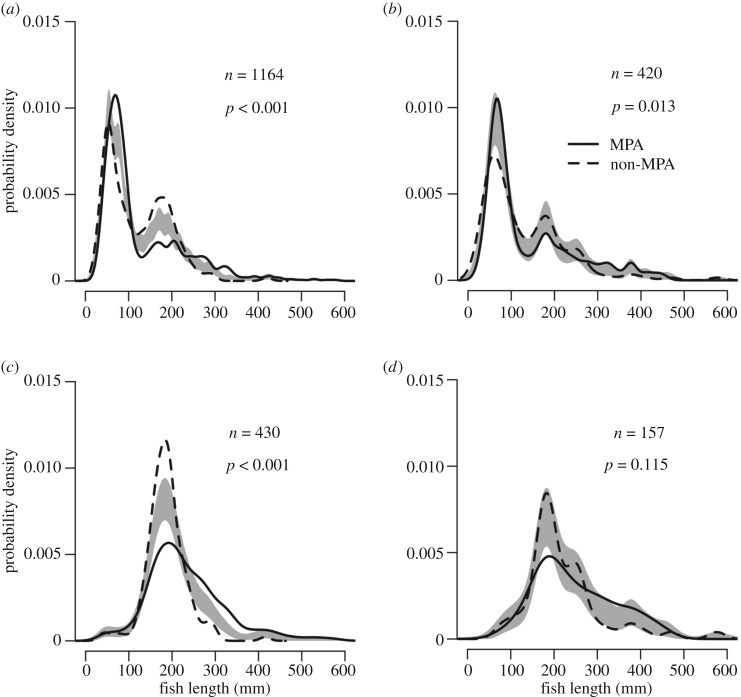
Fish length distributions for all fish species for (*a*) shallow reefs, (*b*) MCEs and for commercially important fish species only for (*c*) shallow reefs and (*d*) MCEs. The grey-shaded area indicates one standard error either side of the null model of no difference in length distribution based on protection. *n* = number of fish.

We tested fish length distributions, comparing inside and outside the MPA, finding that on shallow reefs outside the MPA a greater proportion of the fish are of small (less than 250 mm) body length (figure 6*a*). This pattern is even more extreme when considering only commercially valuable species on unprotected shallow reefs, with a large peak in fish body lengths between 100 and 250 mm, and few individuals bigger than 300 mm (figure 6*c*). Protected shallow reefs also have many fish in the 100–250 mm range, although there are more fish with body lengths in the 250–400 mm range than on unprotected shallow reefs (figure 6*c*). By contrast, on MCEs there are less clear differences between fish length distributions inside and outside the MPA. While there are differences in the length distribution for all recorded MCE fish based on protection, these appear to be driven by differences in the proportion of small fish in the 0–100 mm length range, with larger bodied fish appearing similar (figure 6*b*). When specifically comparing commercially valuable fish on MCEs, we found no difference in the fish length distributions based on protection status (figure 6*d*). In general, we recorded few large fish on reefs at both depths and protection types around Cozumel, with only 10 individuals greater than 500 mm length out of the 2599 recorded fish. These were individuals of: *Caranx latus*, *Mycteroperca bonaci*, *Ocyurus chrysurus*, *Pomacanthus arcuatus* and *Sphyraena barracuda*.

## 4. Discussion

Our results show differences in MCE benthic communities between MPA and non-MPA areas. However, the differences are small, and MCE communities differed less than shallow reef communities based on protection. This supports the idea that MCEs could—by virtue of their depth—receive some protection from adjacent shallow reef disturbance. However, we identified that most hard coral species found on shallow reefs are absent on MCEs. This suggests that MCEs may have limited ability to aid threatened shallow reef hard corals to recover. By contrast, we found 42.5% of fish species recorded on both shallow reefs and MCEs, including many commercially valuable fish species. Our results, therefore, indicate that MCEs have the potential to play a role in supporting shallow reef fish populations.

### 4.1. Differences between inside and outside MPA for shallow reefs and MCEs

We found higher hard coral cover on shallow reefs inside the MPA compared to shallow reefs outside, which were dominated by non-living components (e.g. discarded artificial structures and sand) and sponges. Shallow reef communities inside the MPA naturally exist as a series of built-up reefs separated by patches of sand; therefore, also have a large proportion of non-living benthic cover. Caution must be used when comparing MPA sites with non-protected sites. Without historical data prior to MPA designation, it is not possible to establish whether the observed patterns were directly caused by the MPA. Hence, our results should be seen as a status assessment of current ecological communities inside and outside the MPA, rather than a direct evaluation of MPA impact.

Regardless of protection and location, all observed Cozumel MCEs were continuous reefs with the main benthic cover provided by macroalgae, sponges and gorgonians. Hard corals were present on MCEs, although these were at low abundance. There was no effect of protection on any individual MCE benthic community component surveyed except gorgonians. Gorgonian cover was on average approximately four times higher on sites inside the MPA than outside. It is not clear what drives these patterns, as it has previously been suggested that gorgonians are more resilient to disturbance impacts and other environmental factors than many other reef organisms such as hard corals [51,52]. Interestingly, some sites which clustered close together in the RDA benthic analysis in the shallows also did so on MCEs, for example, outside the MPA Transito Transbordador and Purgatorio, and inside the MPA Palancar Jardines and Herradura. This suggests similar environmental or anthropogenic processes may be driving benthic communities on shallow reefs and MCEs. In addition to being associated with higher hard coral cover in the shallows, both Palancar Jardines and Herradura were associated with higher hard coral cover on MCEs, with Herradura having the highest hard coral coverage we observed on Cozumel MCEs at 5.1 ± 2.0%. In this context, our results would suggest that the disturbance associated with Cozumel town could be affecting benthic communities on MCEs and shallow reefs.

Previous shallow reef surveys at one of our non-MPA study sites, Villa Blanca, have reported large declines in hard coral cover. Here, shallow reef hard coral cover declined from 44% in 1995 to 4% in 2005 [39], which is more severe than declines recorded within the MPA during this time [39]. We recorded hard coral cover at Villa Blanca at less than 1% suggesting that further declines have occurred. This unprotected area is adjacent to Cozumel town with multiple cruise ships, passenger and car ferries passing over and docking adjacent to the reef daily. There is also a large cruise ship terminal which—during construction—appeared to severely affect shallow reefs [29,39].

Total fish biomass, on both shallow reefs and MCEs, was higher within the MPA than outside when aggregating all fish species, and also just commercially valuable species. Four Pomacentridae species had higher biomass inside the MPA area than outside, though none of the commercially valuable fish species that correlated with our PCOA axes had higher biomass inside the MPA. Despite the overall higher fish biomass on sites inside the MPA than outside, Cozumel shallow reef fish biomass within the MPA is already considered low for the region [37]. This suggests that fish populations on shallow sites outside the protected area are even more severely depleted. These shallow reef findings are further supported by the fish length distributions, showing fewer large fish on shallow reefs outside the MPA, particularly those of higher commercial value. This contrasts with fish length distribution comparisons for MCEs, where there was no difference in commercially valuable fish length based on protection status. This could potentially suggest a depth refuge for larger fish on MCEs outside the MPA, or may indicate that commercially valuable fish are depleted across all our survey sites in Cozumel. However, this finding must be treated with caution, as fewer commercially valuable fish were measured on MCEs than on shallow reefs (157 versus 430), reducing power to discern differences based on protection on MCEs. Hence, further work is required to establish whether there are differences in length distributions based on protection on MCEs.

Because of the location of the MPA on the southwest coast and Cozumel town on the northwest coast, MPA and non-MPA sites are separated into two discrete groups along the coastline. We included latitude as the first term in our models, and used Type 1 (sequential) sums of squares. This means that each subsequent model term was fitted after taking account of the previously fitted terms, allowing us to test for protection and depth effects after accounting for any latitude effects. While our protection and depth results are robust to latitudinal effects, we did identify latitudinal patterns. For example, the sites furthest south (Palancar Jardines and Herradura) had higher shallow hard coral cover than the other sites further north inside the MPA and the non-MPA sites. These furthest south sites also had the highest hard coral cover on MCEs, suggesting that factors driving these hard coral cover in the shallows may also be influencing MCEs. Both of these sites are furthest away from Cozumel town, and are the first reefs that currents pass over along the coast of Cozumel [53]. Currents can influence water quality and correlate with both benthic and fish community structure [54,55]. This design seems ideal for the MPA, as it suggests that currents could naturally carry new recruits into the impacted areas. The influence of environmental factors such as current strength and direction in association with recruitment should be investigated in future studies.

### 4.2. Community ecology across shallow reefs to MCEs around Cozumel

All surveyed MCEs were located on steep slopes as extensions of the shallow reef community. This characteristic reduces the light levels available to benthic organisms rapidly with increased depth [12,16]. MCEs had lower hard coral cover than the shallows, which is consistent with previous qualitative observations of MCEs around Cozumel [31,56,57]. For example, Dahlgren [31] observations from the 1980s report that hard coral-dominated reefs ended at approximately 30 m, including at two of our study sites: Colombia and Santa Rosa. Zlatarski [57] surveyed in 1983–1984 finding that scleractinians were rare at increased depths and near-absent below 50 m. Also, Günther [56] surveys from 1987 document deeper slopes in the 40–50 m range dominated by algae with large sponges and octocorals present. They also report small isolated hard coral colonies present of mostly *Helioseris cucullata*, *Porites astreoides* and *Eusmilia fastigiata*. Interestingly, while quantitative data broken down by site and depth are not available from these earlier studies, our results suggest that unlike shallow reefs, MCEs on Cozumel have not changed much in broad benthic composition. We also observed high presence of macroalgae, sponges and octocorals, as well as small colonies of *H. cucullata*. There is one exception to this, Cozumel historically was famed for extensive black coral populations on MCEs which were harvested until the mid-1990s [27,30]. We detected low black coral densities, and recent work has indicated that Cozumel black coral densities have further declined since the mid-1990s [35]. Our results support the idea that MCEs by virtue of their depth have provided some protection from adjacent coastal development for some species, and that the main benthic community that dominates MCEs is macroalgae, sponges and gorgonians.

Surprisingly, we did not find a strong effect of depth on total fish biomass, or commercially valuable fish biomass, despite depth being a key factor structuring the overall fish community. Decreasing fish biomass with increasing depth has been documented on the southern Mesoamerican Reef [26,58], and also at other locations in the Caribbean such as Curaçao [59] and Puerto Rico [60]. It is not clear why we did not detect a larger decline in overall fish biomass with increased depth, though we identified several species of Acanthuridae, Labridae, Pomacentridae and Scaridae that declined on deeper reefs, which is similar to patterns recorded on the southern Mesoamerican Reef [26,58,61]. Patterns of decline in herbivorous fish biomass have been widely observed on MCEs in the western Atlantic [58,60,62], so declines in herbivorous Acanthuridae and Scaridae are not surprising. In addition, we identified two MCE specialist fish species, *Prognathodes aculeatus* and *Chromis insolata* that appear to be important in driving fish community patterns.

Overall, 42.5% of fish species recorded were found on both shallow reefs and MCEs, indicating a high level of species overlap between depths. Of the commercially valuable species we recorded, 39.6% were present at both depths, and 11.3% were only recorded on MCEs. This indicates that MCEs in this study could play a role in supporting commercially important fish species. However, regardless of protection and depth, we found only 10 individual fish greater than 500 mm length out of the 2599 recorded fish, and there was a general absence of large predatory fish from the reefs of Cozumel. Historically, the Mexican Caribbean hosted several grouper spawning aggregation sites, with groupers up to 880 mm in length recorded from the region as recently as the mid-1990s [63]. The absence of large-bodied grouper is consistent with other studies of reefs facing fisheries pressure within the Mesoamerican Reef region. For example, surveys conducted on almost 150 Mesoamerican Reef shallow sites found that large groupers (greater than 400 mm) were scarce—present in only 11% of locations—and far lower than historical length distributions [37,63]. While studies on MCEs on the southern Mesoamerican Reef have revealed increased fish body size on MCEs compared to shallow reefs, suggesting possible refuges, there were still limited numbers of larger predatory fish found [58]. However, other studies have identified that Caribbean MCEs do appear to be acting as refuges for historically overfished large predatory species such as sharks and groupers [60,64].

Recent work has highlighted the refuge role that MCEs can play for invasive lionfish in the Caribbean [65,66], because areas with shallow reef lionfish culling can still leave large lionfish abundances on MCEs [67]. On Cozumel, there is widespread shallow lionfish culling by the recreational dive community and fishers, and as would be expected with sustained culling pressure we did not observe any lionfish on our shallow fish transects. Interestingly however, we only observed two individual lionfish on our MCE transects, one at Villa Blanca and one at Herradura. Therefore, despite MCEs acting as refuges for lionfish from culling in the southern Mesoamerican Reef [67], MCEs on the west coast of Cozumel do not appear to have a similar lionfish refuge role.

### 4.3. Integrating MCEs into current MPA management

Our results show that MCEs contain diverse benthic communities with many fish species—previously reported from shallow reefs—associated with them. We found mixed evidence on whether MCEs are buffered from adjacent coastal development disturbances affecting shallow reefs. However, our results also indicate that MCEs contain unique benthic assemblages and many commercially valuable species (electronic supplementary material, S9) that could potentially be benefiting from existing protection. The whole depth range of reef ecosystems, including MCEs, should be considered when designing and implementing reef management plans [9]. Previous examples imply that where coral reef management is already in place for shallow areas, incorporating MCEs into management plans can be easier, accelerating protection [9].

Overexploitation of shallow reef fisheries combined with new technology has led to fisheries expansion to MCEs [20,68]. We identified low commercially valuable fish biomass and low black coral densities compared to historical records [30,35,63]. Hence, Cozumel MCEs require protection beyond any natural depth refuge effects. More studies are necessary to understand ecological dynamics and the long-term changes occurring on Cozumel upper-MCEs. Given that the Cozumel National Park extends to the 100 m isobath, we recommend MCEs to be recognized in the National Park management plan and promote research, monitoring and protection efforts for these deeper reefs.

## 5. Conclusion

This study provides the first quantitative characterization of MCEs on the west side of Cozumel and compares them with adjacent shallow reefs within and outside of the National Park. We identified differences in benthic communities and fish communities between MCE and shallow sites, and sites inside and outside the MPA, suggesting that MCEs can be affected by adjacent coastal development. Our study highlights the need to integrate MCEs in current reef management plans because they are a continuation of shallow coral reefs containing both unique species and many threatened and commercially valuable shallow reef species.

## Supplementary Material

Study site GPS locations.

## Supplementary Material

Raw benthic data.

## Supplementary Material

Raw fish abundance and biomass data.

## Supplementary Material

Fish length weight conversion and commercial value categories.

## Supplementary Material

Raw fish length data.

## Supplementary Material

ESM 6 Benthic key.csv

## Supplementary Material

ESM 7 R Code.txt

## Supplementary Material

ESM 8 Coral species observed on Cozumel.docx

## Supplementary Material

ESM 9 Fish species observed on Cozumel.docx

## Permission to carry out fieldwork

Permission to carry out fieldwork was granted to E.G. by the Comisión Nacional de Áreas Naturales Protegidas (CONANP) Dirección del Parque Nacional ‘Arrecifes de Cozumel’. Permit no. F00.9/DPNAC/305-16 F00.9.DRPYCM.00778/2016.

## Data accessibility

All raw data and R code for analysis are contained within electronic supplementary material.
