# Supplementary material for: Assessing mesophotic coral ecosystems inside and outside a Caribbean marine protected area
Source: R Soc Open Sci. 2018 Oct 31;5(10):180835. doi: 10.1098/rsos.180835 (PMC6227970; doi:10.1098/rsos.180835)
Supplement: ESM 8 Coral species observed on Cozumel.docx [file rsos180835supp8.docx]

**ESM 8**. Hard coral species (Scleractinia) and black coral species (Antipatharia) observed on shallow reefs (15 m) and MCEs (55 m) at surveyed sites around Cozumel.

| **Order** | **Genus** | **Species** | **Shallow Reef** | **MCE** | **Authority** |
| --- | --- | --- | --- | --- | --- |
| Scleractinia |  |  | - | - |  |
|  | *Diploria* | *labyrinthiformis* | Observed | - | Linnaeus, 1758 |
|  | *Eusmilia* | *fastigiata* | Observed | - | Pallas, 1766 |
|  | *Helioseris* | *cucullata* | - | Observed | Ellis & Solander, 1786 |
|  | *Meandrina* | *meandrites* | Observed |  | Linnaeus, 1758 |
|  | *Mycetophyllia* | *aliciae* | - | Observed | Wells, 1973 |
|  | *Mycetophyllia* | *lamarckiana* | Observed | - | Milne Edwards & Haime, 1848 |
|  | *Orbicella* | *annularis* | Observed | - | Ellis & Solander, 1786 |
|  | *Porites* | *astreoides* | Observed | - | Lamarck, 1816 |
|  | *Porites* | *divaricata* | Observed | - | Le Sueur, 1820 |
|  | *Porites* | *furcata* | Observed | - | Lamarck, 1816 |
|  | *Porites* | *porites* | Observed | - | Pallas, 1766 |
|  | *Siderastrea* | *siderea* | Observed | Observed | Ellis & Solander, 1768 |
|  | *Undaria* | *agaricites* | Observed | Observed | Linnaeus, 1758 |
|  | *Undaria* | *tenuifolia* | Observed | - | Dana, 1848 |
| Antipatharia |  |  |  |  |  |
|  | *Antipathes* | *caribbeana* | - | Observed | Opresko, 1996 |
|  | *Plumapathes* | *Pennacea* | - | Observed | Pallas, 1766 |
