# Supplementary material for: Assessing mesophotic coral ecosystems inside and outside a Caribbean marine protected area
Source: R Soc Open Sci. 2018 Oct 31;5(10):180835. doi: 10.1098/rsos.180835 (PMC6227970; doi:10.1098/rsos.180835)
Supplement: ESM 9 Fish species observed on Cozumel.docx [file rsos180835supp9.docx]

**ESM 9**. Fish species observed on shallow reefs (15 m) and MCEs (55 m) at surveyed sites around Cozumel. Commercially valuable is defined based from Fishbase (see methods and ESM 4).

| Family | Genus | Species | Shallow Reef | MCE | Commercially Valuable | Authority |
| --- | --- | --- | --- | --- | --- | --- |
| Acanthuridae | *Acanthurus* | *bahianus* | Observed | Observed | X | Castelnau, 1855 |
| Acanthuridae | *Acanthurus* | *chirurgus* | Observed | Observed | X | Bloch, 1787 |
| Acanthuridae | *Acanthurus* | *coeruleus* | Observed | Observed | X | Bloch & Schneider, 1801 |
| Balistidae | *Balistes* | *vetula* | Observed | Observed | X | Linnaeus, 1758 |
| Balistidae | *Canthidermis* | *sufflamen* | Observed | - | X | Mitchill, 1815 |
| Balistidae | *Melichthys* | *niger* | Observed | - | X | Bloch, 1786 |
| Balistidae | *Xanthichthys* | *ringens* | Observed | Observed | X | Linnaeus, 1758 |
| Carangidae | *Caranx* | *crysos* | Observed | Observed | - | Mitchill, 1815 |
| Carangidae | *Caranx* | *latus* | Observed | - | X | Agassiz, 1831 |
| Carangidae | *Caranx* | *ruber* | Observed | Observed | X | Bloch, 1793 |
| Chaetodontidae | *Chaetodon* | *capistratus* | Observed | Observed | - | Linnaeus, 1758 |
| Chaetodontidae | *Chaetodon* | *ocellatus* | Observed | Observed | - | Bloch, 1787 |
| Chaetodontidae | *Chaetodon* | *sedentarius* | Observed | Observed | - | Poey, 1860 |
| Chaetodontidae | *Chaetodon* | *striatus* | Observed | Observed | - | Linnaeus, 1758 |
| Chaetodontidae | *Prognathodes* | *aculeatus* | - | Observed | - | Poey, 1860 |
| Grammatidae | *Gramma* | *loreto* | Observed | - | - | Poey, 1868 |
| Haemulidae | *Anisotremus* | *surinamensis* | Observed | - | X | Bloch, 1791 |
| Haemulidae | *Anisotremus* | *virginicus* | Observed | - | X | Linnaeus, 1758 |
| Haemulidae | *Haemulon* | *carbonarium* | Observed | - | X | Poey, 1860 |
| Haemulidae | *Haemulon* | *flavolineatum* | Observed | - | X | Desmarest, 1823 |
| Haemulidae | *Haemulon* | *macrostomum* | - | Observed | X | Günther, 1859 |
| Haemulidae | *Haemulon* | *melanurum* | Observed | - | X | Linnaeus, 1758 |
| Haemulidae | *Haemulon* | *parra* | Observed | - | X | Desmarest, 1823 |
| Haemulidae | *Haemulon* | *plumierii* | Observed | Observed | X | Lacepède, 1801 |
| Haemulidae | *Haemulon* | *sciurus* | Observed | - | X | Shaw, 1803 |
| Haemulidae | *Haemulon* | *steindachneri* | Observed | - | X | Jordan & Gilbert, 1882 |
| Holocentridae | *Holocentrus* | *adscensionis* | - | Observed | X | Osbeck, 1765 |
| Kyphosidae | *Kyphosus* | *sectatrix* | Observed | - | X | Linnaeus, 1758 |
| Labridae | *Bodianus* | *rufus* | Observed | - | X | Linnaeus, 1758 |
| Labridae | *Clepticus* | *parrae* | Observed | - | X | Bloch & Schneider, 1801 |
| Labridae | *Halichoeres* | *bivittatus* | Observed | - | - | Bloch, 1791 |
| Labridae | *Halichoeres* | *garnoti* | Observed | Observed | - | Valenciennes, 1839 |
| Labridae | *Halichoeres* | *maculipinna* | Observed | Observed | - | Müller & Troschel, 1848 |
| Labridae | *Halichoeres* | *pictus* | Observed | - | - | Poey, 1860 |
| Labridae | *Halichoeres* | *radiatus* | - | Observed | X | Linnaeus, 1758 |
| Labridae | *Thalassoma* | *bifasciatum* | Observed | Observed | - | Bloch, 1791 |
| Lutjanidae | *Lutjanus* | *analis* | Observed | - | X | Cuvier, 1828 |
| Lutjanidae | *Lutjanus* | *apodus* | Observed | Observed | X | Walbaum, 1792 |
| Lutjanidae | *Lutjanus* | *buccanella* | Observed | - | X | Cuvier, 1828 |
| Lutjanidae | *Lutjanus* | *griseus* | Observed | - | X | Linnaeus, 1758 |
| Lutjanidae | *Lutjanus* | *mahogoni* | Observed | Observed | X | Cuvier, 1828 |
| Lutjanidae | *Lutjanus* | *synagris* | Observed | - | X | Linnaeus, 1758 |
| Lutjanidae | *Ocyurus* | *chrysurus* | Observed | Observed | X | Bloch, 1791 |
| Malacanthidae | *Malacanthus* | *plumieri* | Observed | - | X | Bloch, 1786 |
| Monacanthidae | *Aluterus* | *scriptus* | Observed | - | - | Osbeck, 1765 |
| Monacanthidae | *Cantherhines* | *pullus* | Observed | - | X | Ranzani, 1842 |
| Mullidae | *Pseudupeneus* | *maculatus* | Observed | - | X | Bloch, 1793 |
| Ostraciidae | *Acanthostracion* | *polygonius* | Observed | - | X | Poey, 1876 |
| Pomacanthidae | *Holacanthus* | *ciliaris* | Observed | Observed | X | Linnaeus, 1758 |
| Pomacanthidae | *Holacanthus* | *tricolor* | Observed | Observed | X | Bloch, 1795 |
| Pomacanthidae | *Pomacanthus* | *arcuatus* | Observed | Observed | X | Linnaeus, 1758 |
| Pomacanthidae | *Pomacanthus* | *paru* | Observed | Observed | X | Bloch, 1787 |
| Pomacentridae | *Abudefduf* | *saxatilis* | Observed | - | - | Linnaeus, 1758 |
| Pomacentridae | *Chromis* | *cyanea* | Observed | Observed | - | Poey, 1860 |
| Pomacentridae | *Chromis* | *insolata* | Observed | Observed | - | Cuvier, 1830 |
| Pomacentridae | *Chromis* | *multilineata* | Observed | - | - | Guichenot, 1853 |
| Pomacentridae | *Microspathodon* | *chrysurus* | Observed | - | - | Cuvier, 1830 |
| Pomacentridae | *Stegastes* | *adustus* | Observed | Observed | - | Troschel, 1865 |
| Pomacentridae | *Stegastes* | *diencaeus* | Observed | - | - | Jordan & Rutter, 1897 |
| Pomacentridae | *Stegastes* | *leucostictus* | Observed | - | - | Müller & Troschel, 1848 |
| Pomacentridae | *Stegastes* | *partitus* | Observed | Observed | - | Poey, 1868 |
| Pomacentridae | *Stegastes* | *planifrons* | Observed | - | - | Cuvier, 1830 |
| Pomacentridae | *Stegastes* | *variabilis* | Observed | - | - | Castelnau, 1855 |
| Scaridae | *Scarus* | *coeruleus* | Observed | - | X | Edwards, 1771 |
| Scaridae | *Scarus* | *iseri* | Observed | Observed | X | Bloch, 1789 |
| Scaridae | *Scarus* | *taeniopterus* | Observed | - | X | Lesson, 1829 |
| Scaridae | *Scarus* | *vetula* | Observed | Observed | X | Bloch & Schneider, 1801 |
| Scaridae | *Sparisoma* | *aurofrenatum* | Observed | Observed | X | Valenciennes, 1840 |
| Scaridae | *Sparisoma* | *chrysopterum* | Observed | Observed | X | Bloch & Schneider, 1801 |
| Scaridae | *Sparisoma* | *rubripinne* | Observed | Observed | X | Valenciennes, 1840 |
| Scaridae | *Sparisoma* | *viride* | Observed | Observed | X | Bonnaterre, 1788 |
| Scorpaenidae | *Pterois* | *volitans* | - | Observed | X | Linnaeus, 1758 |
| Serranidae | *Cephalopholis* | *cruentata* | - | Observed | X | Lacepède, 1802 |
| Serranidae | *Cephalopholis* | *fulva* | Observed | Observed | X | Linnaeus, 1758 |
| Serranidae | *Epinephelus* | *adscensionis* | Observed | - | X | Osbeck, 1765 |
| Serranidae | *Hypoplectrus* | *nigricans* | Observed | - | - | Poey, 1852 |
| Serranidae | *Mycteroperca* | *bonaci* | - | Observed | X | Poey, 1860 |
| Serranidae | *Serranus* | *tigrinus* | Observed | - | - | Bloch, 1790 |
| Sphyraenidae | *Sphyraena* | *barracuda* | Observed | - | X | Edwards, 1771 |
| Tetraodontidae | *Canthigaster* | *rostrata* | Observed | Observed | - | Bloch, 1786 |
